# Dialysis therapy in Brazil: the significance of data from the Brazilian Society of Nephrology in understanding real-life scenarios and its role in shaping effective public policies

**DOI:** 10.1590/2175-8239-JBN-2025-E003en

**Published:** 2025-02-10

**Authors:** Natalia Maria S. Fernandes, Marcus G. Bastos, Fernando A.B. Colugnati

**Affiliations:** 1 Universidade Federal de Juiz de Fora, Departamento de Clínica Médica, Juiz de Fora, MG, Brazil.; 2 SUPREMA, Faculdade de Ciências Médicas e da Saúde de Juiz de Fora, Juiz de Fora, MG, Brazil.; 3 Centro Universitário Governador Ozanam Coelho (UNIFAGOC), Ubá, MG, Brazil.; 4 Universidade Federal de Juiz de Fora, Faculdade de Medicina, Departamento de Internato, Juiz de Fora, MG, Brazil.

Clinical epidemiology is the science that allows us to make inferences and predictions about individual patients by quantifying clinical events and the factors associated with them. This is achieved by observing and monitoring similar patients using robust scientific methods that allow accurate interpretations, generalizations, and conclusions. Chronic kidney disease (CKD) is a major public health problem, primarily due to its rising prevalence and the high costs associated with its treatment. Understanding the epidemiology of CKD is crucial for shaping effective public policies. It is estimated that 850 million individuals worldwide are affected by CKD, a figure that exceeds the number of people living with diabetes mellitus or human immunodeficiency virus (HIV)^1^. In 1999, the Brazilian Society of Nephrology (BSN) launched the BSN Census to gather data from dialysis units across Brazil. At that time, the term “census” was appropriate, as the response rate was an impressive 99.8%^2^. However, since then, the response rates of the annual surveys conducted by BSN have decline each year, making it increasingly challenging to draw general conclusions from the data. In the study “Brazilian Dialysis Survey 2023”, the authors used a new methodology to improve inferences, despite the low response rate by clinics^3^. The voluntary response rate in the study was 37.5% and involved a simple random sample that allowed for a more accurate estimate, a design that in a way meets all the assumptions of even the most basic statistical methods. In addition, adjustments were made to sampling weights to maintain regional representativeness and minimize the impact of non-response, which occurs even in this type of sampling. The results of the random sample indicate an increase in prevalence and incidence of 775 ppm and 251 ppm, respectively, consistent with the national trend observed in previous years, carried out only by voluntary samples. When we analyze prevalence rates at a regional level (Figure 2 of reference 3), we uncover significant variations, particularly in the Central-West and northern regions, which likely indicate previous selection bias^3^. This phenomenon is also apparent in the voluntary samples and the additionally analyzed variables. These comparisons often subject to major uncertainties, especially concerning clinical profiles that are predominantly linked to private healthcare. Such results may stem from selection bias associated with voluntary responses, making it challenging to reach definitive conclusions without consulting secondary data sources, conducting literature reviews, and incorporating the clinical expertise of survey coordinators. While the effort and confidence behind these findings are commendable, they lack the rigorous scientific foundation expected in clinical epidemiology.

## Future Possibilities

Obtaining accurate data is an ongoing challenge, but BSN’s dedication to enhancing data quality provides the opportunity to obtain auxiliary databases and develop new methodologies. Embracing a probabilistic approach, as emphasized in the article, is vital for accurately assessing prevalence and incidence. This strategy will significantly improve the collection of clinical variables, which is crucial for advancing our understanding in this field.

It is imperative to recognize the significant advancements in data acquisition. One underutilized resource is the Department of Information Technology of the Unified Health System (DATASUS), an open-access database rich with information, including the procedures financed by the SUS. With the SUS covering over 80% of dialysis procedures in Brazil, using DATASUS could greatly enhance the accuracy of patient estimates and their outcomes. By tapping into this valuable data source, we can refine our estimates and improve overall healthcare insights. The integration of these databases is becoming more effective than ever by advanced pre-processing methods that facilitate probabilistic linkages. Tools available in statistical packages like *Microdatasus* ^4^ can significantly enhance this process, paving the way for more accurate and insightful data analysis.

The acquisition of individual information through a unified digital platform can significantly transform the way clinics respond to patient needs while adhering to the General Data Protection Law (LGPD). This valuable data not only enhances the quality of voluntary responses, but can also be effectively processed later using post-stratification techniques that leverage crucial information from the initial sample design, ultimately leading to improved patient care and clinic performance.

Ensuring the quality of the BSN Survey data is vital, as it forms the basis for numerous studies on patients receiving dialysis treatment. Additionally, these data serve as a key resource for shaping effective public policies, as their accuracy and reliability allow them to be used to direct public policies.
