# Supplementary material for: Dialysis therapy in Brazil: the significance of data from the Brazilian Society of Nephrology in understanding real-life scenarios and its role in shaping effective public policies
Source: J Bras Nefrol. 2025 Feb 10;47(1):e2025E003. doi: 10.1590/2175-8239-JBN-2025-E003en (PMC11813189; doi:10.1590/2175-8239-JBN-2025-E003en)
Supplement: Translation

# Translation: Terapia dialítica no Brasil: a importância dos dados da Sociedade Brasileira de Nefrologia para o entendimento do cenário da vida real e no direcionamento das políticas públicas

**DOI:** 10.1590/2175-8239-JBN-2025-E003pt

**Authors:** Natalia Maria S. Fernandes, Marcus G. Bastos, Fernando A.B. Colugnati

**Affiliations:** 1 Universidade Federal de Juiz de Fora, Departamento de Clínica Médica, Juiz de Fora, MG, Brasil.; 2 SUPREMA, Faculdade de Ciências Médicas e da Saúde de Juiz de Fora, Juiz de Fora, MG, Brasil.; 3 Centro Universitário Governador Ozanam Coelho (UNIFAGOC), Ubá, MG, Brasil.; 4 Universidade Federal de Juiz de Fora, Faculdade de Medicina, Departamento de Internato, Juiz de Fora, MG, Brasil.

A epidemiologia clínica é a ciência que possibilita inferências e predições sobre pacientes individuais quantificando eventos clínicos e fatores associados aos mesmos, a partir da observação e o seguimento de pacientes similares, utilizando métodos científicos sólidos que permitam interpretações, generalizações e conclusões com exatidão e precisão aceitáveis. A doença renal crônica (DRC) é um grande problema de saúde pública por sua prevalência crescente e altos custos de tratamento. Conhecer adequadamente a epidemiologia da DRC é de suma importância, principalmente para guiar políticas públicas. Estima-se que 850 milhões de pessoas apresentem DRC em todo o mundo, um número superior ao de pessoas que vivem com diabetes *mellitus* ou o vírus da síndrome da imunodeficiência humana^1^. Em 1999, a Sociedade Brasileira de Nefrologia (SBN) introduziu o Censo SBN para coletar dados das unidades de diálise do Brasil. À época, o termo censo foi adequado, já que a taxa de resposta foi de 99,8%^2^. Desde então, a SBN vem mantendo todo o ano um levantamento (*survey*) que sofre anualmente com a diminuição na taxa de resposta, dificultando a realização de generalizações. No estudo “Brazilian Dialysis Survey 2023”, utilizam uma nova metodologia para melhorar as inferências, ainda que com baixa taxa de resposta das clínicas^3^. A taxa de resposta voluntária no estudo foi de 37,5% e foi associada à amostra aleatória simples que possibilitou a realização de estimativa mais precisa, delineamento que de certa forma atende a todas as suposições mesmo dos métodos estatísticos mais básicos. Além disso, foram realizados ajustes nos pesos amostrais para manter a representatividade regional e minimizar o impacto da não resposta, que, mesmo neste tipo de amostragem, ocorre. Os resultados da amostra aleatória apontam crescimento na prevalência e na incidência de 775 pmp e 251 pmp, respectivamente, compatíveis com a tendência nacional observada nos anos anteriores, realizadas somente por amostras voluntárias. No entanto, ao olharmos essas prevalências ao nível regional (Figura 2 da referência 3), observamos comportamentos diferenciados principalmente na região Centro-Oeste e Norte, que possivelmente demonstram os vieses de seleção do passado^3^. E isso continua sendo refletido no caso das amostras voluntárias, principalmente nas demais variáveis analisadas. Essas comparações serão sempre grandes pontos de interrogação, como os perfis de clínica mais voltados para a saúde privada. Esses achados podem ocorrer por um viés de seleção ocasionado pela resposta voluntária, ou seja, fica difícil concluir sem fontes de dados secundários, revisão de literatura e da prática clínica dos coordenadores deste levantamento. Apesar do esforço e da confiança, não é um método científico robusto, como definido na epidemiologia clínica.

## Possibilidades Futuras

Dados reais são sempre um desafio, e o compromisso da SBN em fornecer dados cada vez mais aprofundados e precisos é um chamado à utilização de bases de dados auxiliares e de novas metodologias. Manter uma abordagem probabilística, como a descrita no artigo, é crucial a partir de agora para as prevalências e incidências, visando aprimorar a coleta de variáveis clínicas, especialmente.

É importante salientar a melhora na aquisição de dados. Uma das fontes que pode ser mais bem explorada é o Departamento de Informática do Sistema Único de Saúde (DATASUS), o qual é uma base de dados de acesso livre que contém, entre outros dados, procedimentos pagos pelo SUS. O SUS é responsável pelo pagamento de mais de 80% dos procedimentos dialíticos no Brasil. A utilização do DATASUS como fonte de dados para melhorar a estimativa do número de pacientes e o desfecho deles poderia auxiliar na precisão dos dados. A integração dessas bases de dados está cada vez melhor, utilizando-se métodos de pré-processamento que facilitam*linkages*probabilísticos, inclusive já disponíveis em pacotes estatísticos como o*Microdatasus* ^4^.

A aquisição de informações individuais por meio de uma plataforma digital unificada conforme a lei geral de proteção de dados (LGPD) pode ser uma ferramenta valiosa para aprimorar a resposta das clínicas. Esses dados, que seriam uma melhoria nas respostas voluntárias, podem ser trabalhados posteriormente por meio de técnicas de pós-estratificação que utilizam informações essenciais para o delineamento amostral já realizado.

A melhoria da qualidade dos dados do *Survey* da SBN é crucial, uma vez que esses dados são, com frequência, usados como base para outros estudos que utilizam pacientes em tratamento dialítico, além de serem utilizados como ferramenta para direcionar políticas públicas.
